# Development and internal validation of a nomogram for predicting 90-day poor functional outcome after mechanical thrombectomy in very elderly patients: A retrospective cohort study

**DOI:** 10.1097/MD.0000000000050265

**Published:** 2026-08-21

**Authors:** Dewang Pan, Zhiguo Li, Junfeng Zhao, Liyu Xia, Huaiyu Niu, Yang Zheng, Shuangxu Tan, Yu Li, Guoliang Li, Ying Liu

**Affiliations:** a Department of Neurology, Siping Central People’s Hospital, Siping, Jilin, China.

**Keywords:** acute ischemic stroke, mechanical thrombectomy, nomogram, prognosis, very elderly

## Abstract

Mechanical thrombectomy (MT) is increasingly performed in very elderly patients with acute ischemic stroke, but individualized in-hospital prognostic assessment after treatment remains limited, particularly after early postprocedural events have become clinically apparent. This study aimed to develop and internally validate a nomogram for predicting 90-day poor functional outcomes in very elderly patients after MT. We retrospectively enrolled very elderly patients (aged ≥ 80 years) with acute ischemic stroke who underwent MT at a single center between January 1, 2020 and August 1, 2025. Poor functional outcome was defined as a modified Rankin Scale score of 3 to 6 at 90 days. Candidate predictors, including baseline, peri-procedural, and early postprocedural variables, were screened using univariable logistic regression and incorporated into a multivariable model. Because complete separation was observed for collateral status, the final model was constructed using Firth penalized logistic regression. Model discrimination was assessed using the area under the receiver operating characteristic curve, calibration was evaluated by the Brier score, calibration intercept, and calibration slope, and internal validation was performed with bootstrap resampling. Clinical utility was assessed using decision curve analysis. A total of 214 patients were included, of whom 80 (37.4%) had poor outcomes and 134 (62.6%) had good outcomes at 90 days. The final nomogram incorporated 6 predictors: National Institutes of Health Stroke Scale score at admission, diabetes mellitus, puncture-to-recanalization time, postoperative pneumonia, symptomatic intracranial hemorrhage (sICH), and poor collateral status. The model showed good discrimination, with an area under the curve of 0.874 (bootstrap 95% confidence interval, 0.811–0.929). Calibration was acceptable, with a Brier score of 0.112, a calibration intercept of 0.057, and a calibration slope of 1.090. Decision curve analysis demonstrated a meaningful net benefit across threshold probabilities ranging from 0.01 to 0.99. We developed and internally validated a nomogram for predicting 90-day poor functional outcomes in very elderly patients after MT. This model may assist in-hospital prognostic stratification, risk communication, and discharge planning in already treated patients after early postprocedural complications have been identified or excluded. However, it was not designed for pretreatment decision-making or for estimating the treatment effect of endovascular therapy.

## 1. Introduction

Clinical epidemiological studies have shown that stroke remains one of the leading causes of death and disability worldwide. In the Global Burden of Disease 2021 analysis, stroke accounted for 7.3 million deaths, 93.8 million prevalent cases, and 11.9 million incident cases globally.^[1]^ In China, national surveillance data showed 28.76 million prevalent cases, 3.94 million incident cases, and 2.19 million stroke-related deaths, and a 2020 nationwide survey reported an age-standardized prevalence of 2.61%, an incidence of 505.2 per 100,000, and a mortality of 343.4 per 100,000.^[2]^ Importantly, recent Chinese burden analyses further indicate that the stroke burden is strongly age-related, with higher prevalence in older adults and higher mortality in the elderly. Ischemic stroke is the most prevalent kind of stroke. The incidence and percentage of acute ischemic stroke (AIS) among older persons have been rising annually as China steadily transitions to an aging society.^[3]^ Mechanical thrombectomy (MT) is an endovascular reperfusion procedure in which catheters and thrombectomy devices are advanced into an occluded intracranial artery to achieve rapid recanalization, and it has become a standard treatment option for selected patients with AIS caused by large-vessel occlusion.^[4,5]^ For older patients who are good candidates and have experienced an AIS, MT has become more and more common in many countries in recent years due to advancements in endovascular procedures and devices. As a result, an increasing proportion of patients with AIS who are very old (over 80) are choosing MT.

When compared to the general population of patients who have had an AIS, elderly people often have a lower overall physiological reserve. Treatment in this population continues to be particularly difficult for stroke centers that have only a limited amount of experience. The question of how to effectively choose acceptable senior candidates and consistently predict clinical outcomes following MT in older patients has emerged as a significant clinical challenge and a research hotspot.^[6,7]^ Logistic regression is a standard and widely accepted method for developing individualized clinical prediction models when predictors are defined at an appropriate time point and model performance is properly evaluated. Nomograms provide a graphical representation of regression-based models and may facilitate bedside risk estimation.^[8]^ However, most pretreatment prediction approaches are based primarily on baseline demographic, clinical, or imaging variables available before endovascular therapy (EVT) and therefore cannot fully capture important determinants that become apparent only during or after thrombectomy, such as puncture-to-recanalization efficiency, symptomatic intracranial hemorrhage (sICH), postoperative pneumonia, and early postprocedural recovery trajectory. Accordingly, prognostic assessment performed after thrombectomy may provide more clinically actionable information for in-hospital risk stratification, family counseling, and discharge planning in already treated very elderly patients.

Recent studies also indicate that functional outcomes remain guarded in this population. In patients aged ≥ 80 years undergoing MT, a very poor 90-day outcome has been reported in 56.5% of cases, and multicenter data in nonagenarians have highlighted a high risk of futile recanalization despite successful reperfusion. Moreover, nationwide data from the United States showed that, among EVT-treated patients, those aged ≥ 80 years less often achieved functional independence at discharge and more often died or were discharged to hospice care than younger patients. These findings further underscore the clinical vulnerability and prognostic heterogeneity of very elderly patients after thrombectomy, thereby strengthening the rationale for individualized postprocedural risk prediction in this age group.^[9,10]^ Taken together, these data support the need for a dedicated post-thrombectomy prognostic tool rather than relying solely on pretreatment selection metrics in this particularly vulnerable population.

To date, few pragmatic prognostic tools have been developed specifically for very elderly patients undergoing MT. Given the increasing use of thrombectomy in this age group and the substantial heterogeneity in postprocedural recovery, there is a clinical need for tools that can support individualized risk assessment during hospitalization. Therefore, we conducted this study to develop and internally validate a nomogram for estimating the risk of 90-day functional disability in very elderly patients (≥ 80 years) who have already undergone MT. Importantly, this study was designed as a prognostic modeling study within a treated cohort rather than as a comparative effectiveness analysis of EVT versus non-EVT management. Because several candidate predictors reflect peri-procedural or early postprocedural events, the intended use of the model is in-hospital prognostic stratification and risk communication in already treated patients after key early postprocedural complications have been identified or excluded, rather than pretreatment decision-making.

## 2. Materials and methods

This study is reported in accordance with the Transparent Reporting of a Multivariable Prediction Model for Individual Prognosis or Diagnosis statement for prediction model development and internal validation.

Ethics statement: This retrospective single-center study was approved by the local institutional ethics committee (Approval No. LLWYH-2019-04-007), and the requirement for informed consent was waived because of the retrospective design.

### 2.1. Study population

We conducted a retrospective evaluation of the clinical data of very elderly patients who had suffered an AIS and had undergone MT at the stroke center of our hospital between the dates of January 1, 2020 and August 1, 2025.

Inclusion criteria: age ≥ 80 years; symptom onset within 24 hours; large-vessel occlusion confirmed by intraoperative digital subtraction angiography (DSA); National Institutes of Health Stroke Scale (NIHSS) score > 6 on admission; and meeting the indications for endovascular intervention.

Exclusion criteria: intracerebral hemorrhage or subarachnoid hemorrhage confirmed by computed tomography (CT) or magnetic resonance imaging (MRI); severe abnormalities in coagulation parameters; active advanced solid or hematologic malignancy with an expected life expectancy of ≤ 6 months, to minimize confounding from severe non-stroke-related short-term mortality; a definite history of psychiatric illness or severe cognitive impairment precluding reliable follow-up; and missing clinical data. A complete-case analysis was performed; patients with missing candidate-predictor or outcome data were excluded from model development. Patients included in the final analytic cohort had complete 90-day outcome data and complete data for predictors used in the final model.

### 2.2. Treatment

For patients presenting within the intravenous thrombolysis window (symptom onset ≤ 6 hours), the patient and family members were asked whether intravenous thrombolysis should be performed. If intravenous thrombolysis was requested, thrombolysis was administered first, and endovascular thrombectomy was performed subsequently if the thrombolytic response was unsatisfactory. If the patient and/or family declined intravenous thrombolysis within the time window and requested direct endovascular thrombectomy, MT was performed directly.

Intravenous thrombolysis protocol: Patients presenting within the intravenous thrombolysis window received bridging therapy when indicated according to institutional stroke protocols and patient/family preference. Intravenous thrombolysis was administered using standard approved regimens, followed by repeat vascular assessment and MT when appropriate. In selected patients between 4.5 and 6 hours from symptom onset, urokinase-based thrombolysis was used according to local practice and eligibility.

Following the completion of intravenous thrombolysis, a repeat vascular evaluation was carried out making use of brain MRI in conjunction with perfusion-weighted imaging and magnetic resonance angiography, head-and-neck CT angiography (CTA) in conjunction with CT perfusion, or direct DSA. Bridging therapy was then provided based on the reassessment findings. For patients beyond the intravenous thrombolysis window, emergent head-and-neck CTA + computed tomography perfusion or brain MRI + perfusion-weighted imaging +  magnetic resonance angiograph was performed as appropriate; if large-vessel occlusion was confirmed, MT was performed.

MT procedure: Conscious sedation with analgesia was routinely used. General anesthesia was selected if intraoperative agitation could not be controlled or if other indications for general anesthesia were present. All patients underwent arterial access using a modified Seldinger technique via femoral artery puncture. If severe lower-extremity atherosclerotic occlusive disease was present, brachial or radial artery access was used instead. After successful access, a 6F sheath was inserted, and complete cerebral angiography was performed to reconfirm the occlusion site and collateral circulation. Once the target vessel was identified, a microcatheter was positioned proximal to the lesion. A microwire was advanced across the occlusion, followed by the advancement of the microcatheter. After confirming entry into the true lumen distal to the occlusion by contrast injection through the microcatheter, a stent retriever was deployed across the occluded segment and allowed to integrate with the thrombus for approximately 5 to 10 minutes. The assistant then withdrew the microcatheter and stent retriever under continuous negative pressure using a 50-mL syringe. Control angiography was performed thereafter. If reperfusion did not reach modified thrombolysis in cerebral infarction (mTICI) grade 2b or higher, adjunctive aspiration thrombectomy using an intermediate catheter (e.g., Navien) was performed depending on the thrombus burden during the initial retrieval. If mTICI 2b or higher was achieved after the first pass, angiography was repeated after 10 minutes; if no flow slowing was observed, the procedure was terminated.

If more than 3 thrombectomy passes were performed and reperfusion remained below mTICI 2b, rescue strategies were considered, including intra-arterial medication, balloon angioplasty, and/or stent implantation. After the procedure, the puncture site was closed by suturing or a vascular closure device as appropriate. Postoperatively, patients received individualized medical management, including antiplatelet therapy, anticoagulation, neuroprotective and supportive treatments, and measures to improve cerebral circulation when indicated. All thrombectomy procedures were performed by attending surgeons with a professional title of associate chief physician or above.

### 2.3. Outcome definition and model development/validation

At the end of the first ninety days, the modified Rankin Scale (mRS) was utilized to evaluate the functional outcome. A good outcome (mRS 0–2) or a poor outcome (mRS 3–6) was assigned to each patient, and the patients were grouped accordingly.

The 90-day mRS was obtained using a standardized, structured assessment via outpatient clinic visits, structured telephone interviews, or WeChat-based follow-up (voice/video/text), depending on patient preference and feasibility. Because of the retrospective real-world design, centralized blinded adjudication was not available; however, outcome assessment was performed using a standardized structured mRS follow-up approach in routine clinical practice. Outcome data were complete for all included patients, with no loss to follow-up.

The univariable logistic regression method was initially utilized to assess the potential factors. Subsequently, variables that exhibited possible connections and/or clinical relevance were incorporated into a multivariable logistic regression model to discover independent predictors of poor outcomes at the 90-day mark. To facilitate individualized risk estimation, a regression-based nomogram was constructed in R based on the final multivariable model, in accordance with established clinical prediction modeling principles. Rather than being used for pre-procedural treatment selection, the model is designed for in-hospital prognostic estimation during the postprocedural course, rather than for treatment selection at admission or immediately after thrombectomy.

**Figure 1.**
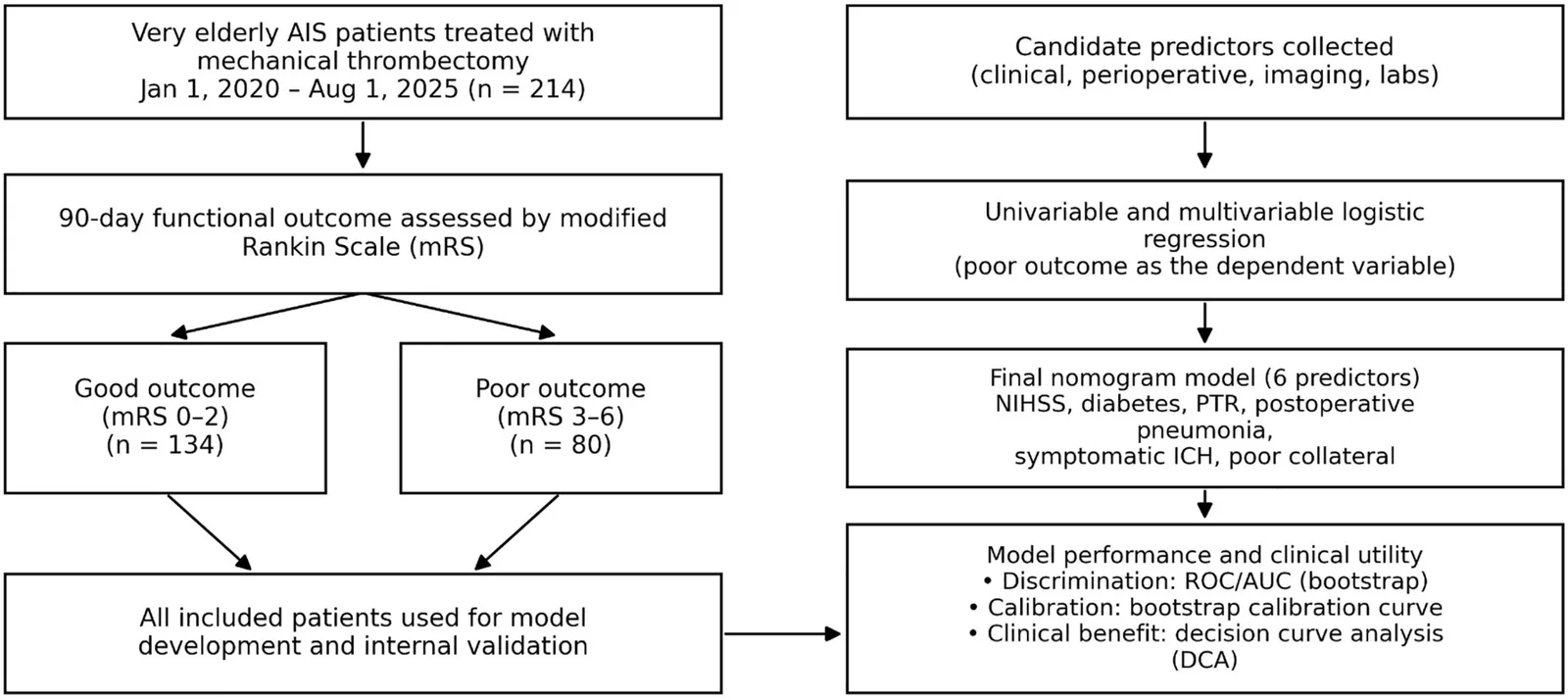
Study workflow and cohort stratification. Very elderly patients (≥ 80 years) with AIS treated with mechanical thrombectomy between January 1, 2020 and August 1, 2025 were included (n = 214). Ninety-day functional outcome was assessed using the mRS and categorized as good outcome (mRS 0–2; n = 134) or poor outcome (mRS 3–6; n = 80). Candidate predictors were evaluated by logistic regression, and a 6-factor nomogram was developed and internally validated using bootstrap resampling; model performance and clinical utility were assessed by ROC/AUC, calibration, and DCA. AIS = acute ischemic stroke, AUC = area under the curve, DCA = decision curve analysis, mRS = modified Rankin Scale, ROC = receiver operating characteristic.

The receiver operating characteristic (ROC) analysis (area under the curve [AUC]) was utilized to quantify the discrimination performance of the model. Internal validation and calibration performance were examined with bootstrap resampling, and clinical utility was evaluated with decision curve analysis (DCA) to estimate net benefit over clinically relevant threshold probabilities. In the DCA, net benefit was estimated across threshold probabilities from 0.01 to 0.99. Both of these evaluations were carried out to determine overall clinical utility. Figure 1 diagrammatically depicts the overall workflow of the investigation.

### 2.4. Outcome measures and assessment

In addition to laboratory parameters and perioperative and imaging-related indicators, the observed variables included all clinically relevant patient data, such as age, sex, and stroke-related risk factors (such as hypertension, diabetes mellitus, atrial fibrillation, and history of smoking and alcohol consumption). More severe neurological abnormalities are indicated by higher scores on the NIHSS scale, which runs from 0 to 42. Higher scores on the mRS, which has a range of 0 to 6, imply less functional independence; a score of 6 denotes death. Restoring blood flow at the initially occluded segment to mTICI grade ≥ 2b following stent-retriever thrombectomy was considered successful recanalization. According to the American Society for Interventional and Therapeutic Neuroradiology/Society for Interventional Radiology (ASITN/SIR) system, collateral circulation was graded on a scale of 0 to 4. Poor collaterals were graded 0 to 1, moderate collaterals were graded 2, and good collaterals were graded 3 to 4.Two skilled neurointerventionalists used the ASITN/SIR scale to judge collateral status on DSA; discrepancies were settled by consensus.According to widely accepted ECASS-II criteria, sICH within 24 hours after MT was defined as any intracranial hemorrhage temporally associated with neurological impairment (an increase of ≥ 4 points in NIHSS) or death. In accordance with routine in-hospital diagnostic procedure, the treating team diagnosed postoperative pneumonia occurring within 7 days after MT or during the index hospitalization based on compatible clinical symptoms (e.g., fever, leukocytosis/leukopenia, purulent sputum) and new/progressive infiltrates on chest imaging.

### 2.5. Statistical analysis

The poor functional outcome at 90 days was the primary endpoint, which was defined as a mRS score between 3 and 6. The univariable logistic regression method was initially utilized in order to assess the potential factors. To find independent predictors of poor outcomes, variables that showed possible connections and/or were judged clinically relevant were later incorporated into a multivariable logistic regression model. Candidate predictors considered at model entry included baseline demographic and vascular risk factors, admission stroke severity, infarct location, workflow/procedural characteristics, collateral status, early postprocedural complications, and selected laboratory indices; all predictors were entered using their original coding, with 1 degree of freedom for binary/continuous variables and k-1 degrees of freedom for multicategory variables. Potential nonlinearity for continuous predictors, particularly admission NIHSS and PTR, was examined using restricted cubic splines with 3 knots. Because no material deviation from linearity was observed and spline terms did not meaningfully improve model fit, continuous predictors were retained as linear terms on their original scales in the final model. No hyperparameter tuning was required for Firth penalized logistic regression. The optimal operating cutoff probability was defined as the threshold that maximized the Youden index on the ROC curve. In the present study, diabetes mellitus and PTR were retained in the final multivariable model despite *P* values > .05 because their inclusion was guided not only by statistical significance but also by prespecified clinical relevance, prior evidence, and overall modeling strategy. Diabetes has been consistently associated with worse outcomes after recanalization therapies in AIS, and PTR is a clinically meaningful workflow variable directly reflecting reperfusion efficiency and the time-to-reperfusion paradigm. Given the modest sample size and the aim of building a clinically interpretable prognostic tool for already treated very elderly patients, we prioritized the preservation of these clinically important predictors in the final model rather than excluding them solely on the basis of arbitrary significance thresholds. Bootstrap resampling was used to estimate internal model performance and calibration, but no explicit global shrinkage factor or post hoc coefficient adjustment was applied to the final reported nomogram. Accordingly, the presented model should be interpreted as an unshrunk derivation model, and its apparent performance may still reflect some optimism despite bootstrap validation.

In order to produce finite and reliable coefficient estimates for the final model, bias-reduced (Firth penalized) logistic regression was employed. This was done because the collateral-status indicator (ASITN/SIR 0–1) revealed complete separation in this dataset. An individual risk estimation nomogram was produced by using the final set of predictors as the basis for the development of the nomogram.

In order to assess the discrimination capabilities of the model, the apparent area under the ROC curve (AUC) was utilized, together with bootstrap-based confidence intervals. Bootstrap resampling (*B* = 2000) was used for internal validation and calibration plotting. Calibration was summarized using apparent calibration metrics, including the Brier score, calibration intercept, and calibration slope. In order to assess the net benefit over clinically relevant threshold probabilities, clinical utility was evaluated through the use of DCA. In the DCA, net benefit was estimated across threshold probabilities from 0.01 to 0.99. Because no explicit coefficient shrinkage was applied, these performance estimates are reported as derivation-sample estimates and should be interpreted cautiously with respect to model transportability.

## 3. Results

### 3.1. Cohort characteristics and 90-day outcomes

A total of 214 very elderly patients (age ≥ 80 years) who underwent MT between January 1, 2020 and August 1, 2025 were included. At 90 days, 134 patients (62.6%) achieved a good functional outcome (mRS 0–2), and 80 (37.4%) had a poor outcome (mRS 3–6). Baseline clinical characteristics are summarized in Table 1.

**Table 1:** Baseline clinical characteristics according to 90-day prognosis after mechanical thrombectomy in elderly AIS patients.

Item	Good prognosis (n = 134)	Poor prognosis (n = 80)	*χ*^2^/*t*	*P*
Age (yrs)	86.94 ± 3.74	86.05 ± 3.08	1.793	.074
BMI (kg/m^2)	21.76 ± 2.34	21.82 ± 2.53	−0.161	.872
Systolic BP (mm Hg)	150.11 ± 30.35	148.50 ± 31.09	0.374	.709
Diastolic BP (mm Hg)	87.09 ± 11.55	87.22 ± 10.74	−0.084	.933
NIHSS score at admission	16.14 ± 4.10	20.62 ± 4.68	−7.339	**< .001**
Sex (male/female)	91/43	43/37	3.707	.054
Previous stroke	71 (53.0%)	40 (50.0%)	0.079	.778
Atrial fibrillation history	44 (32.8%)	43 (53.8%)	8.236	**.004**
Diabetes history	74 (55.2%)	63 (78.8%)	11.037	**< .001**
Hypertension history	107 (79.9%)	62 (77.5%)	0.055	.814
Smoking	67 (50.0%)	59 (73.8%)	10.710	**.001**
Drinking	65 (48.5%)	30 (37.5%)	2.033	.154
Bridging therapy	57 (42.5%)	37 (46.2%)	0.150	.699
Infarct location			5.158	**.023**
Anterior circulation	94 (70.1%)	43 (53.8%)		
Posterior circulation	40 (29.9%)	37 (46.2%)		
TOAST classification			3.171	.366
Large-artery atherosclerosis	82 (61.2%)	49 (61.3%)		
Cardioembolism	47 (35.1%)	31 (38.8%)		
Other	3 (2.2%)	0 (0.0%)		
Undetermined	2 (1.5%)	0 (0.0%)		

Data are presented as mean ± standard deviation for continuous variables and n (%) for categorical variables, unless otherwise indicated; sex is presented as male/female counts.

AIS = acute ischemic stroke, BMI = body mass index, BP = blood pressure, n = number of patients, NIHSS = National Institutes of Health Stroke Scale, TOAST = Trial of Org 10172 in Acute Stroke Treatment.

### 3.2. Baseline clinical characteristics

The neurological abnormalities that were present at the time of admission were more severe in individuals who had poor outcomes after 90 days (NIHSS: 20.62 ± 4.68 versus 16.14 ± 4.10; *P* < .001). In the group with poor outcomes, the frequencies of atrial fibrillation were greater (53.8% compared to 32.8%; *P* = .004), diabetes was higher (78.8% compared to 55.2%; *P* < .001), and smoking was higher (73.8% compared to 50.0%; *P* = .001). In addition, there were differences in the location of the infarct across the groups (*P* = .023), with patients who had poorer outcomes having a higher percentage of posterior-circulation infarcts. There were no significant differences in terms of age, gender, body mass index, blood pressure, hypertension, previous stroke, drinking history, bridging therapy, or TOAST categorization (all *P* > .05; Table 1).

### 3.3. Peri-procedural and imaging-related indicators

Table 2 displays indicators relating to imaging and peri-procedural procedures. Patients with poor outcomes had longer in-hospital workflow and procedure durations, such as the time from admission to puncture (80.07 ± 23.65 vs 69.33 ± 24.84 minutes; *P* = .002) and the time from puncture to reperfusion (82.39 ± 29.86 vs 70.93 ± 22.24 minutes; *P* = .002), than those in the good-outcome group. The poor-outcome group’s collateral status was significantly worse (ASITN/SIR: 1.70 ± 0.46 vs 3.55 ± 0.75; *P* < .001). Patients with poor outcomes were more likely to experience symptomatic cerebral bleeding (33.8% vs 5.2%; *P* < .001) and postoperative pneumonia (48.8% vs 20.1%; *P* < .001). Onset-to-admission time, number of thrombectomy passes, device choice, thrombus escape, primary stent implantation, and effective recanalization rate did not differ significantly across the groups (all *P* > .05; Table 2).

**Table 2:** Perioperative and imaging-related indicators according to 90-day prognosis.

Item	Good prognosis (n = 134)	Poor prognosis (n = 80)	*χ*^2^/*t*	*P*
Onset-to-admission time (min)	496.58 ± 56.26	510.61 ± 52.16	−1.813	.071
Admission-to-puncture time (min)	69.33 ± 24.84	80.07 ± 23.65	−3.116	**.002**
Puncture-to-reperfusion time (PTR, min)	70.93 ± 22.24	82.39 ± 29.86	−3.198	**.002**
Number of thrombectomy passes	2.74 ± 0.66	2.80 ± 0.62	−0.670	.504
Collateral circulation grade (ASITN/SIR)	3.55 ± 0.75	1.70 ± 0.46	19.907	**< .001**
Stent used for thrombectomy			0.506	.477
Nico RECO stent	69 (51.5%)	46 (57.5%)		
Medtronic Solitaire FR stent	65 (48.5%)	34 (42.5%)		
Thrombus escape	11 (8.2%)	7 (8.8%)	0.000	1.000
Primary stent implantation	22 (16.4%)	17 (21.2%)	0.494	.482
Successful recanalization	129 (96.3%)	74 (92.5%)	0.789	.375
Postoperative pneumonia	27 (20.1%)	39 (48.8%)	17.894	**< .001**
Symptomatic intracranial hemorrhage	7 (5.2%)	27 (33.8%)	28.406	**< .001**

Data are presented as mean ± standard deviation for continuous variables and n (%) for categorical variables.

ASITN/SIR = American Society for Interventional and Therapeutic Neuroradiology/Society for Interventional Radiology, mTICI = modified Thrombolysis in Cerebral Infarction, n = number of patients, PTR = puncture-to-reperfusion time.

### 3.4. Laboratory indicators

Laboratory indicators are summarized in Table 3. Compared with patients with a good outcome, those with a poor outcome had higher D-dimer levels (0.81 ± 0.13 vs 0.75 ± 0.12 mg/L; *P* = .002) and higher CK-MB (60.96 ± 6.26 vs 57.89 ± 6.79 U/L; *P* = .001), whereas INR (1.15 ± 0.32 vs 1.31 ± 0.44; *P* = .006) and total CK (437.24 ± 71.44 vs 458.95 ± 66.91 U/L; *P* = .026) were lower. Other measured laboratory parameters did not differ significantly between groups (all *P* > .05; Table 3).

**Table 3:** Laboratory indicators according to 90-day prognosis.

Item	Good prognosis (n = 134)	Poor prognosis (n = 80)	*t*	*P*
Glucose (mmol/L)	8.93 ± 1.10	9.04 ± 1.83	−0.524	.601
Prothrombin time (PT, s)	15.15 ± 1.75	15.31 ± 1.92	−0.624	.534
Fibrinogen (FIB, g/L)	4.24 ± 0.21	4.23 ± 0.26	0.376	.707
Thrombin time (TT, s)	16.41 ± 2.54	16.92 ± 2.22	−1.515	.131
D-dimer (mg/L)	0.75 ± 0.12	0.81 ± 0.13	−3.217	**.002**
International normalized ratio (INR)	1.31 ± 0.44	1.15 ± 0.32	2.789	**.006**
B-type natriuretic peptide (BNP, pg/mL)	247.47 ± 59.58	262.28 ± 64.12	−1.711	.089
Calcium (mmol/L)	2.08 ± 0.31	2.06 ± 0.46	0.312	.755
Sodium (mmol/L)	140.07 ± 38.14	135.15 ± 38.92	0.907	.365
Potassium (mmol/L)	4.08 ± 0.63	4.01 ± 0.60	0.732	.465
WBC (×10^9/L)	10.68 ± 2.45	10.80 ± 2.82	−0.312	.755
Platelets (×10^9/L)	102.99 ± 9.11	103.74 ± 9.34	−0.581	.562
RBC (×10^12/L)	4.32 ± 0.49	4.41 ± 0.35	−1.522	.129
Hemoglobin (g/L)	137.03 ± 40.18	138.72 ± 39.36	−0.300	.765
LDH (U/L)	256.94 ± 52.69	249.85 ± 65.53	0.869	.386
AST (U/L)	243.39 ± 49.88	247.04 ± 56.81	−0.492	.623
CK (U/L)	458.95 ± 66.91	437.24 ± 71.44	2.240	**.026**
CK-MB (U/L)	57.89 ± 6.79	60.96 ± 6.26	−3.289	**.001**
cTnI (ng/mL)	0.15 ± 0.05	0.15 ± 0.05	−0.117	.907

Data are presented as mean ± standard deviation.

AST = aspartate aminotransferase, BNP = B-type natriuretic peptide, CK = creatine kinase, CK-MB = creatine kinase-MB, cTnI = cardiac troponin I, FIB = fibrinogen, INR = international normalized ratio, LDH = lactate dehydrogenase, n = number of patients, PT = prothrombin time, RBC = red blood cell count, TT = thrombin time, WBC = white blood cell count.

### 3.5. Independent predictors and nomogram development

**Figure 2.**
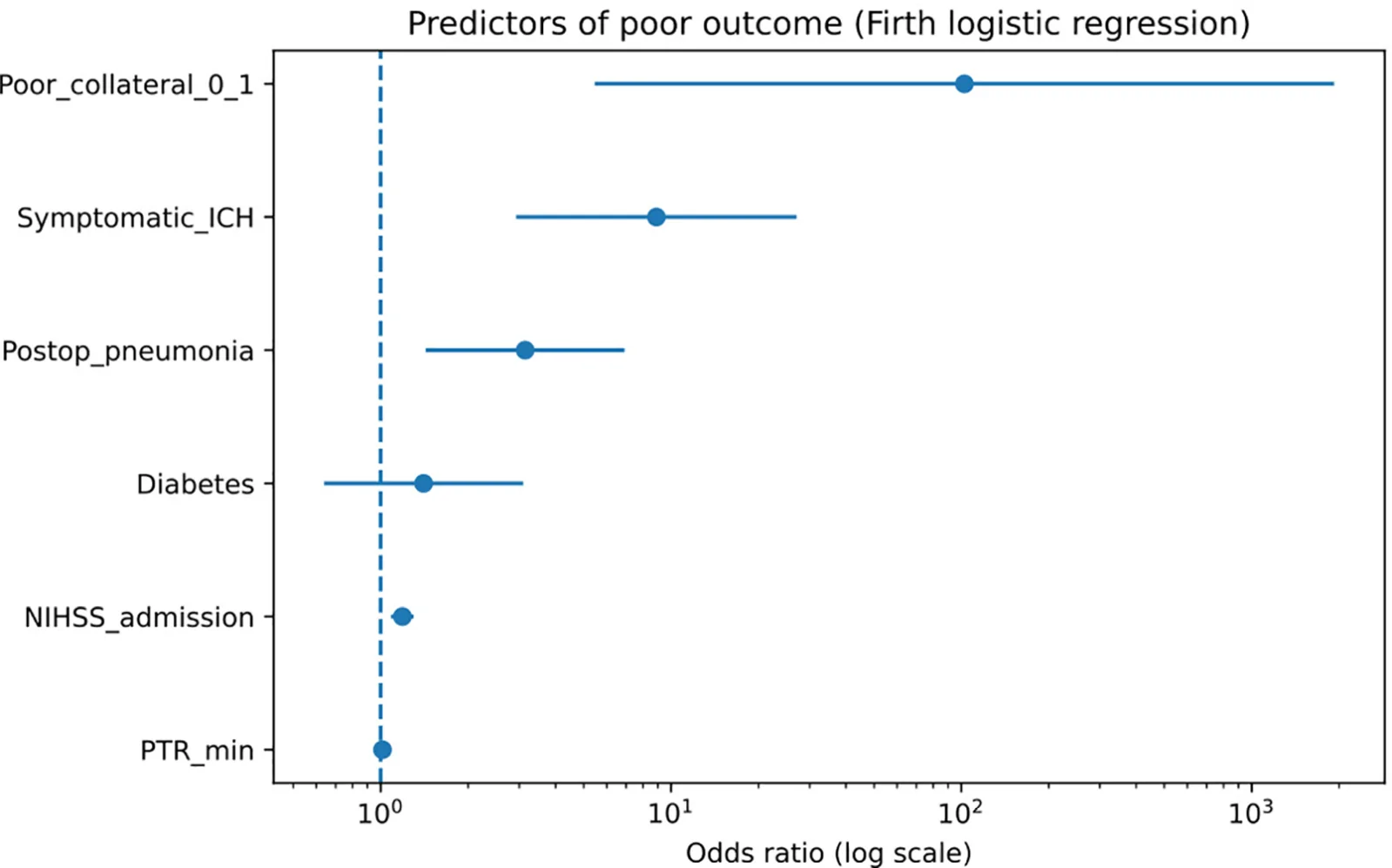
Independent predictors of 90-day poor outcome after thrombectomy. Forest plot of ORs and 95% CIs from the final bias-reduced (Firth penalized) logistic regression model. The vertical dashed line indicates OR = 1. ASITN/SIR = American Society for Interventional and Therapeutic Neuroradiology/Society for Interventional Radiology, CI = confidence interval, NIHSS = National Institutes of Health Stroke Scale, OR = odds ratio, PTR = puncture-to-recanalization time, sICH = symptomatic intracranial hemorrhage.

Variables associated with 90-day outcomes in univariable analyses and/or considered clinically relevant were entered into multivariable modeling. Given complete separation for poor collateral status (ASITN/SIR 0–1), the final model was estimated using bias-reduced (Firth penalized) logistic regression. Six independent predictors were retained: NIHSS score at admission, diabetes, PTR, postoperative pneumonia, sICH, and poor collateral status (Fig. 2; Table 4). The full specification of the final prediction model, including the intercept and regression coefficients, is provided in Table S1, Supplemental Digital Content 1. Diabetes and PTR were retained despite not reaching conventional statistical significance in the final model because both variables were considered clinically important a priori and were supported by previous literature on outcomes after recanalization therapy; retaining them was intended to preserve clinically meaningful information and avoid an overly significance-driven model reduction strategy.

**Table 4:** Coefficients of the final prediction model (Firth logistic regression).

Predictor	*β*	SE	OR	OR (95% CI)	*P* value
NIHSS at admission	0.1719	0.0455	1.188	1.086–1.299	**.0002**
Diabetes	0.3401	0.4025	1.405	0.638–3.093	.3981
Puncture-to-recanalization time (min)	0.0138	0.0081	1.014	0.998–1.030	.0862
Postoperative pneumonia	1.1451	0.4019	3.143	1.430–6.908	**.0044**
Symptomatic intracranial hemorrhage	2.1856	0.5676	8.896	2.925–27.058	**.0001**
Poor collateral (ASITN/SIR 0–1)	4.6289	1.4959	102.404	5.458–1921.497	**.0020**

ASITN/SIR = American Society for Interventional and Therapeutic Neuroradiology/Society for Interventional Radiology, CI = confidence interval, n = number of patients, NIHSS = National Institutes of Health Stroke Scale, OR = odds ratio, SE = standard error, β = regression coefficient.

**Figure 3.**
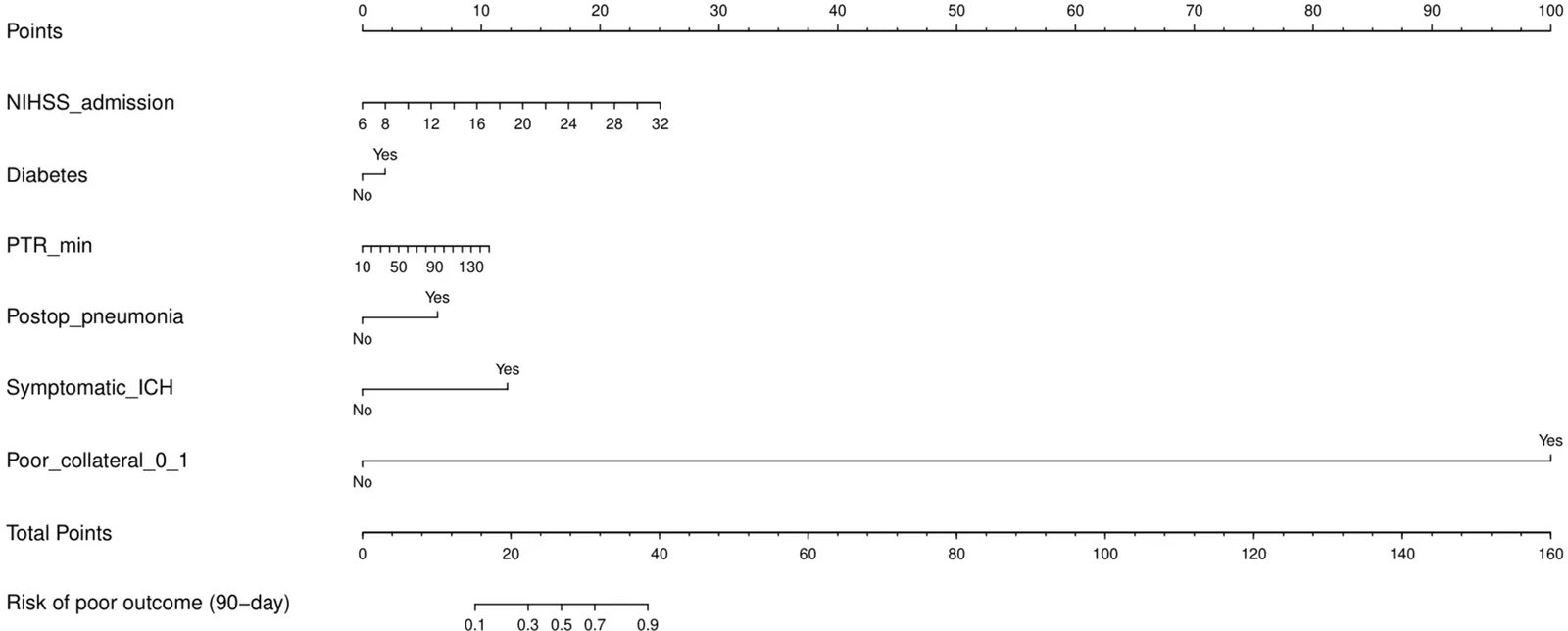
Nomogram for individualized prediction of 90-day poor functional outcome (mRS 3–6). For each predictor, draw a vertical line to the “Points” axis to obtain the corresponding score; sum all scores to obtain “Total Points,” then project to the bottom scale to estimate the probability of poor outcome at 90 days. ASITN/SIR = American Society for Interventional and Therapeutic Neuroradiology/Society for Interventional Radiology, mRS = modified Rankin Scale, NIHSS = National Institutes of Health Stroke Scale, PTR = puncture-to-recanalization time, sICH = symptomatic intracranial hemorrhage.

A nomogram was constructed based on the final model to enable individualized estimation of 90-day poor outcome risk (Fig. 3).

### 3.6. Model discrimination

**Figure 4.**
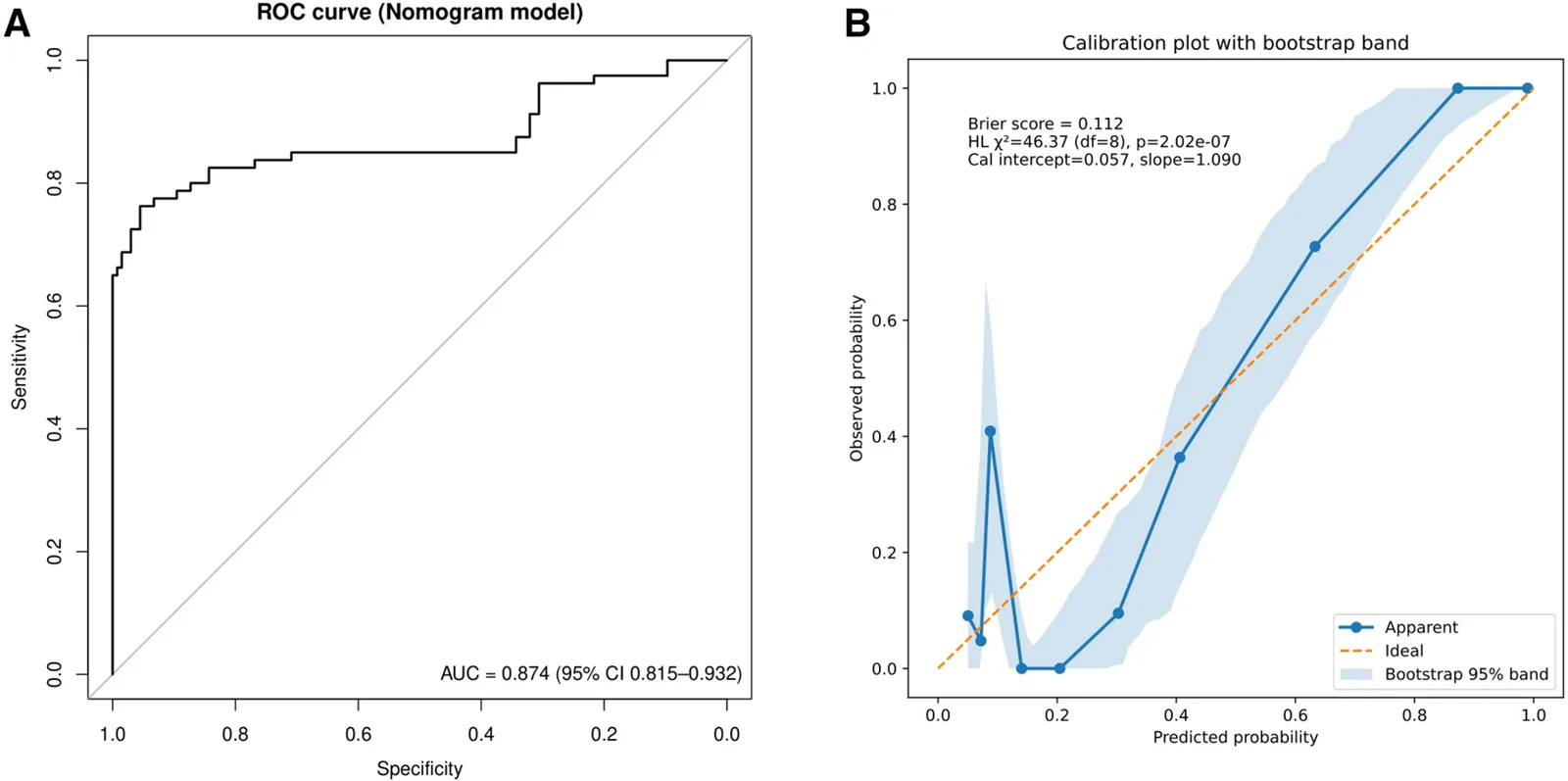
Discrimination and calibration of the nomogram. (A) ROC curve for the nomogram with the apparent AUC and bootstrap 95% CI; (B) calibration plot with bootstrap 95% band comparing predicted versus observed probabilities; the diagonal dashed line represents ideal calibration. AUC = area under the curve, CI = confidence interval, ROC = receiver operating characteristic.

For predicting a poor 90-day outcome, the model showed strong apparent discrimination, with an AUC of 0.874 (bootstrap 95% confidence interval 0.811–0.929; Table S2, Supplemental Digital Content 2). Sensitivity was 0.7625, specificity was 0.9552, and there were 61 true positives, 6 false positives, 19 false negatives, and 128 genuine negatives at the ideal cutoff probability of 0.468, which was derived from ROC analysis by maximizing the Youden index (Table S2, Supplemental Digital Content 2). Figure 4A displays the ROC curve, whereas Figure S1, Supplemental Digital Content 3 provides the precision-recall curve.

### 3.7. Calibration and clinical utility

**Figure 5.**
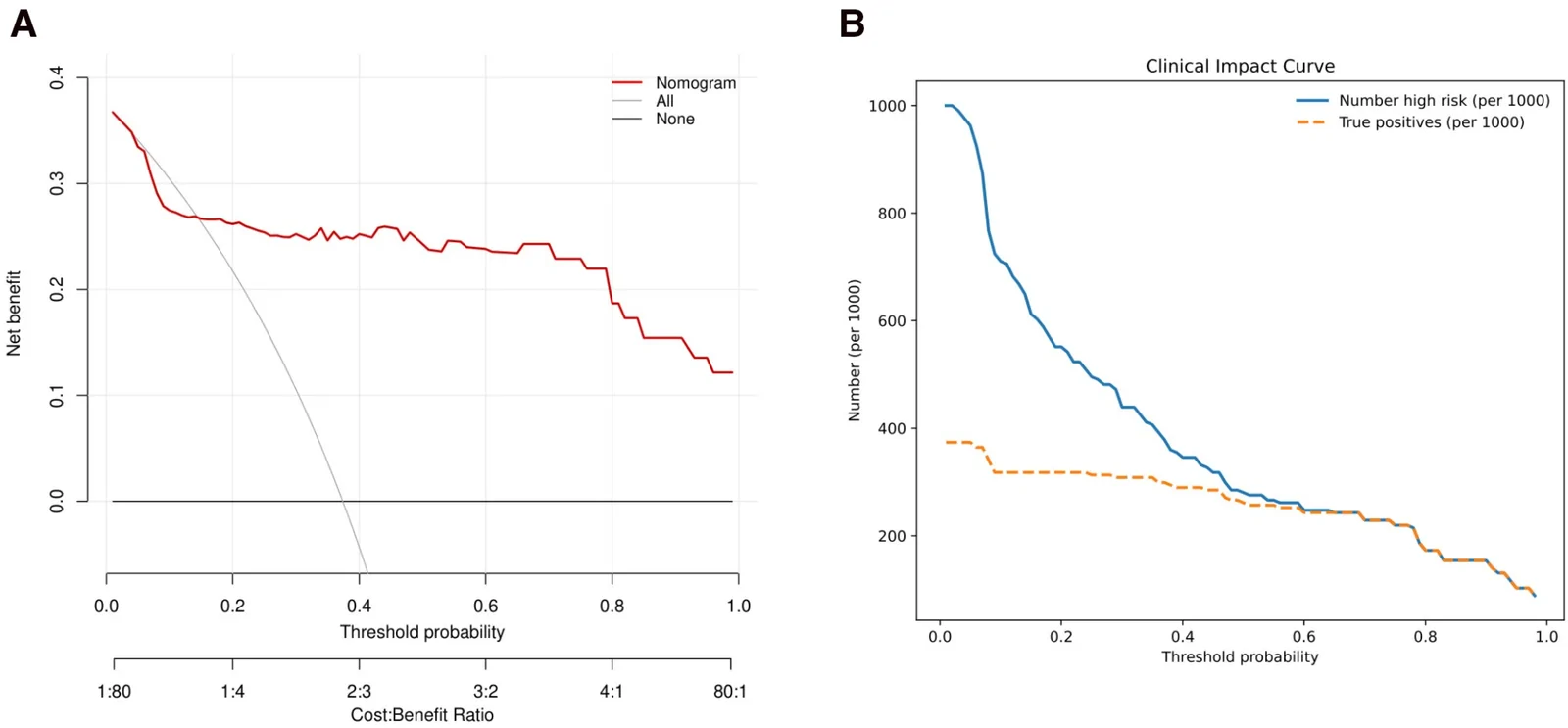
Clinical utility of the nomogram. (A) DCA comparing the net benefit of the nomogram with treat-all and treat-none strategies across threshold probabilities from 0.01 to 0.99; (B) CIC showing the number classified as high risk (per 1000) and the corresponding number of true positives at each threshold probability. CIC = clinical impact curve, DCA = decision curve analysis.

Calibration assessment showed acceptable agreement between predicted and observed risks (Fig. 4B), and an alternative bootstrap calibration presentation is shown in Figure S2, Supplemental Digital Content 4. Apparent calibration statistics showed a Brier score of 0.112, a calibration intercept of 0.057, and a calibration slope of 1.090 (Table S3, Supplemental Digital Content 5), whereas bootstrap resampling was used for internal validation and calibration plotting. When compared to treat-all and treat-none methods, DCA showed a clinically significant net benefit across threshold probabilities from 0.01 to 0.99 (Table S4, Supplemental Digital Content 6; Fig. 5A). Figure S3, Supplemental Digital Content 7 offers an alternate DCA representation. Figure 5B displays the corresponding clinical impact curve. Because the final coefficients were not explicitly shrunk, these calibration results should be interpreted as apparent internal performance rather than fully optimism-corrected final-model performance.

### 3.8. Risk stratification

Patients were stratified into quartiles based on predicted risk (able S5, Supplemental Digital Content 8). Observed event rates increased monotonically across quartiles, from 18.5% in Q1 to 96.3% in Q4, supporting the model’s ability to separate patients into clinically distinct risk groups.

## 4. Discussion

### 4.1. Principal findings

We developed and internally validated a prognostic nomogram to estimate the likelihood of a 90-day poor functional outcome (mRS 3–6) in this cohort of very elderly patients (≥80 years) who were receiving MT between the years 2020 and 2025. The final (Firth penalized) model retained 6 predictors: admission NIHSS, diabetes, PTR, postoperative pneumonia, sICH, and poor collateral status (ASITN/SIR 0–1). Because the model includes peri-procedural and early postprocedural factors, it is best suited for in-hospital prognostic stratification and counseling after the occurrence or exclusion of early postprocedural complications, rather than for pretreatment triage or immediate postprocedural decision-making. Both the nomogram and the DCA indicated that there was a meaningful net benefit across threshold probabilities from 0.01 to 0.99. The nomogram demonstrated robust apparent discrimination (AUC = 0.874; bootstrap 95% confidence interval 0.811–0.929) and acceptable apparent calibration (Brier score of 0.112; calibration slope of 1.090).

### 4.2. Interpretation of key predictors

The identified predictors reflect both baseline stroke severity and potentially modifiable peri-procedural or postprocedural factors, highlighting several actionable targets for outcome improvement in this vulnerable population. In particular, although diabetes and PTR did not meet the conventional *P* < .05 threshold in the final multivariable model, both were intentionally retained because they represent clinically plausible and previously reported determinants of outcome after endovascular reperfusion. We therefore interpreted the final model from a prognostic and clinical-utility perspective rather than using statistical significance alone as the criterion for variable retention.

### 4.3. Admission NIHSS and infarct burden

The most reliable indicator of poor outcome was a higher NIHSS score at the time of admission. This was most likely due to a larger infarct core and/or more extensive involvement of the eloquent cortex.In this context, the salvageable penumbra may be limited, and even successful recanalization may not translate into functional independence if reperfusion occurs after irreversible injury has developed.^[11–16]^ These findings emphasize the importance of rapid evaluation, appropriate imaging-based selection, and minimizing time to reperfusion.

### 4.4. Diabetes and metabolic vulnerability

Diabetes mellitus was retained in the final model and showed an unfavorable direction of effect, consistent with the concept that hyperglycemia can exacerbate ischemic injury through increased anaerobic glycolysis, acidosis, mitochondrial dysfunction, and oxidative stress. In addition, hyperglycemia has the potential to compromise the integrity of the blood–brain barrier and lead to an increased risk of hemorrhagic transformation, which is especially deleterious in the aged population.^[17–19]^ Therefore, peri-procedural glucose management that is extremely stringent while yet being safe may be clinically meaningful.

### 4.5. PTR and the time-to-reperfusion paradigm

PTR captures the efficiency of endovascular workflow after arterial access and serves as a surrogate for the time required to reestablish perfusion. In our cohort, prolonged PTR was associated with worse outcomes, aligning with the well-established time-to-reperfusion principle: delayed reperfusion reduces the probability of penumbral salvage and may amplify reperfusion injury. In practice, PTR can be shortened by optimizing device strategy, team coordination, and rescue-therapy thresholds, especially in complex atherosclerotic anatomy that is common in the very elderly.

### 4.6. Postoperative pneumonia and secondary systemic insults

The statistical analysis revealed that postoperative pneumonia was a significant predictor of poor functional outcome (odds ratio 3.14), highlighting the significance of stroke-associated pneumonia as a potentially preventable consequence. As people get older, their pulmonary reserves decrease, and their immune systems become dysfunctional. This can result in hypoxemia, systemic inflammation, and decreased cerebral oxygen delivery, which can exacerbate neuronal injury and delay recovery.^[20–23]^ There is a possibility that this risk can be reduced through the implementation of standardized preventative bundles, mobilization, routine dysphagia screening, and early airway protection in high-risk patients.

### 4.7. sICH and therapeutic trade-offs

sICH carried a marked increase in the odds of poor outcome (odds ratio 8.90), which is clinically intuitive given its direct parenchymal injury and the subsequent need to withhold or delay antithrombotic therapy, potentially worsening ischemic risk. This vicious cycle may be more pronounced in very elderly patients with fragile microvasculature and comorbidities.^[24–26]^ Careful blood pressure control, individualized antithrombotic timing, and judicious rescue strategies may reduce hemorrhagic complications.

### 4.8. Collateral status as a determinant of tissue survival

Poor collaterals (ASITN/SIR 0–1) showed the largest effect size in the model, reflecting the fundamental role of collateral circulation in sustaining penumbral perfusion before and during reperfusion therapy. Good collaterals can attenuate early ischemic injury and extend the therapeutic window, while poor collaterals predispose patients to rapid infarct growth and worse outcomes. This finding supports systematic collateral assessment during the initial evaluation and may help contextualize prognosis and guide the intensity of peri-procedural and early postprocedural management in very elderly patients.

### 4.9. Model performance and risk stratification

Beyond identifying risk factors, the nomogram provides individualized probability estimates that may support communication and decision-making at the bedside. At the optimal cutoff probability (0.468), the model achieved high specificity (0.955) with moderate sensitivity (0.763), favoring identification of patients at truly high risk. Risk-quartile stratification further demonstrated clear separation: the observed poor-outcome rate increased from 18.5% in the lowest quartile to 96.3% in the highest quartile, indicating clinically meaningful discrimination across risk strata.

### 4.10. Clinical utility and implementation considerations

In the examination of decision curves, it was found that there was a net benefit across threshold probabilities from 0.01 to 0.99. These findings suggest that the model may provide useful prognostic context during hospitalization. However, because some predictors are early postprocedural complications that are already clinically established when the model is applied, its main value lies in risk stratification, clinician-family communication, and discharge-level prognosis estimation rather than in directing specific therapeutic interventions.

### 4.11. Strengths and limitations

This study has several strengths. It focused specifically on very elderly patients, a clinically important yet still relatively understudied subgroup in thrombectomy practice, and it developed an interpretable bedside model using variables that are generally available during routine in-hospital care. The model also underwent bootstrap-based internal validation and showed favorable apparent discrimination and acceptable apparent calibration in the derivation cohort.

Several limitations should, however, be emphasized. First, this was a retrospective single-center study, which introduces the possibility of selection bias and limits generalizability. Second, this study was designed as a prognostic analysis among patients who had already undergone MT, not as a treatment-effect study of EVT versus non-EVT management. Accordingly, the absence of an age-matched non-EVT control group precludes any inference regarding the comparative effectiveness of thrombectomy in very elderly patients, and the model should not be interpreted as evidence for or against offering EVT. Third, some potentially important imaging markers related to infarct burden and tissue viability, such as infarct core volume, alberta stroke program early CT score, or perfusion mismatch, were not uniformly available in standardized form and therefore were not incorporated into the final model. As a result, the present nomogram may not fully capture the contribution of baseline ischemic injury severity. Fourth, premorbid functional status is highly relevant in very elderly stroke patients. Pre-stroke mRS was not routinely available in a standardized form in this retrospective cohort and therefore could not be used as an inclusion criterion or incorporated into the model; future studies should account for premorbid disability more explicitly in very elderly patients. Fifth, the exclusion of patients with severe cognitive impairment that precluded reliable follow-up may have introduced selection bias and may limit generalizability to the broader very elderly stroke population. Sixth, collateral status was derived from intra-procedural DSA using the ASITN/SIR system, and the model may therefore require recalibration before application in centers that rely primarily on CTA-based collateral assessment. Seventh, poor collaterals exhibited complete separation in this dataset, necessitating Firth penalized regression; although this approach yields finite estimates, the corresponding effect sizes should still be interpreted cautiously. Finally, a complete-case analysis was used, and patients with missing data were excluded from the analytic cohort, which may further affect model transportability to settings with different data-completeness profiles. External validation in independent multicenter cohorts, with model recalibration if necessary, will be required before routine clinical use. Eighth, although bootstrap resampling was used for internal validation, no explicit shrinkage factor or coefficient adjustment was applied to the final model. Therefore, some residual optimism in apparent discrimination and calibration cannot be excluded, and recalibration and/or shrinkage may be required when the model is tested in independent external cohorts. In addition, the risk of overfitting may be amplified in a derivation cohort of modest size relative to the number and complexity of candidate predictors, even when bootstrap validation and Firth penalization are used. Furthermore, the transportability of the model may be challenged by between-center differences in patient case mix, premorbid functional status, collateral assessment modality, imaging selection protocols, anesthesia strategy, peri-procedural workflow, rescue treatment preferences, and poststroke complication management. These factors may alter both baseline event rates and predictor–outcome relationships, meaning that the present nomogram should be viewed as a center-specific derivation model that will likely require external validation, calibration updating, and possibly coefficient revision before use in other populations or practice environments.

### 4.12. Future directions

External validation should ideally include centers with different referral structures, imaging workflows, thrombectomy techniques, and poststroke care pathways to more rigorously evaluate model transportability and the need for updating across practice environments.

Future work should externally validate this nomogram in multicenter cohorts with diverse workflow settings and imaging protocols and evaluate prospective clinical impact when the tool is used to guide care pathways. Augmenting the model with advanced imaging markers and developing an online calculator or mobile application may further facilitate clinical adoption.

## 5. Conclusion

In this single-center cohort of very elderly patients (≥ 80 years) who underwent MT, we developed and internally validated a 6-factor nomogram for estimating the risk of 90-day poor functional outcome. The model, which incorporated admission NIHSS, diabetes, PTR, postoperative pneumonia, sICH, and poor collateral status, showed good apparent discrimination and acceptable apparent calibration in the derivation cohort. Its most appropriate role is as an in-hospital prognostic tool for already treated patients once major early postprocedural complications have been established, to support prognostic stratification, clinician-family communication, and discharge planning. However, this model was not designed to estimate the treatment effect of EVT, should not be used to guide whether thrombectomy should be offered, and must be interpreted in light of unmeasured factors such as infarct burden and premorbid functional status. External validation and further refinement in multicenter datasets are required before broader clinical implementation.

## Author contributions

**Conceptualization:** Dewang Pan.

**Data curation:** Dewang Pan.

**Investigation:** Zhiguo Li.

**Methodology:** Zhiguo Li, Huaiyu Niu, Yang Zheng, Yu Li, Guoliang Li.

**Project administration:** Zhiguo Li, Huaiyu Niu, Yang Zheng, Shuangxu Tan, Yu Li, Ying Liu.

**Resources:** Zhiguo Li, Junfeng Zhao, Liyu Xia, Huaiyu Niu, Shuangxu Tan, Guoliang Li, Ying Liu.

**Software:** Junfeng Zhao, Liyu Xia, Shuangxu Tan.

**Supervision:** Junfeng Zhao, Liyu Xia.

**Validation:** Junfeng Zhao.

**Visualization:** Junfeng Zhao.

**Writing – original draft:** Junfeng Zhao.

**Writing – review & editing:** Junfeng Zhao.

















## References

[1] Feigin VL Abate MD Abate YH. Global, regional, and national burden of stroke and its risk factors, 1990–2021: a systematic analysis for the Global Burden of Disease Study 2021. Lancet Neurol. 2024;23:973–1003. 39304265 10.1016/S1474-4422(24)00369-7 PMC12254192

[2] Tu WJ Wang LD. China stroke surveillance report 2021. Mil Med Res. 2023;10:33. 37468952 10.1186/s40779-023-00463-x PMC10355019

[3] Li X Jin T Bai C Wang X Zhang H Zhang T. Analysis of stroke burden in China from 1990 to 2021 and projections for the next decade. Neuroepidemiology. 2025;59:505–16. 39510046 10.1159/000542487

[4] Liu L Li Z Zhou H. Chinese Stroke Association guidelines for clinical management of ischaemic cerebrovascular diseases: executive summary and 2023 update. Stroke Vasc Neurol. 2023;8:e3. 38158224 10.1136/svn-2023-002998 PMC10800268

[5] Adcock AK Schwamm LH Smith EE. Trends in use, outcomes, and disparities in endovascular thrombectomy in US patients with stroke aged 80 years and older compared with younger patients. JAMA Netw Open. 2022;5:e2215869. 35671055 10.1001/jamanetworkopen.2022.15869 PMC9175073

[6] Ippen FM Schregel K Ungerer M Feisst M Ringleb PA Gumbinger CK. Outcomes in elderly patients undergoing endovascular thrombectomy in association with premorbid Rankin Scale scores. Front Neurol. 2024;15:1418415. 39022738 10.3389/fneur.2024.1418415 PMC11252042

[7] Xue J Zhang XG Zhang D. Predictors of very poor outcome after mechanical thrombectomy in older patients with acute ischemic stroke. World Neurosurg. 2024;185:e1224–9. 38514033 10.1016/j.wneu.2024.03.060

[8] D’Anna L Merlino G Romoli M. Predictors of futile recanalization in nonagenarians treated with mechanical thrombectomy: a multi-center observational study. J Neurol. 2024;271:4925–32. 38753228 10.1007/s00415-024-12428-8 PMC11319431

[9] Collins GS Reitsma JB Altman DG Moons KGM. Transparent reporting of a multivariable prediction model for individual prognosis or diagnosis (TRIPOD): the TRIPOD statement. BMJ. 2015;350:g7594. 25569120 10.1136/bmj.g7594

[10] Steyerberg EW. Clinical Prediction Models: A Practical Approach to Development, Validation, and Updating. 2nd ed. Springer; 2019.

[11] Sun D Guo X Nguyen TN. Alberta stroke program early computed tomography score, infarct core volume, and endovascular therapy outcomes in patients with large infarct: a secondary analysis of the ANGEL-ASPECT trial. JAMA Neurol. 2024;81:30–8. 38010691 10.1001/jamaneurol.2023.4430 PMC10682939

[12] Yogendrakumar V Campbell BC Johns HT. Association of ischemic core hypodensity with thrombectomy treatment effect in large core stroke: a secondary analysis of the SELECT2 randomized controlled trial. Stroke. 2025;56:1366–75. 40151939 10.1161/STROKEAHA.124.048899

[13] Winkelmeier L Kniep H Thomalla G. Arterial collaterals and endovascular treatment effect in acute ischemic stroke with large infarct: a secondary analysis of the TENSION trial. Radiology. 2025;314:e242401. 39998372 10.1148/radiol.242401

[14] Sadeler A Finitsis S Olivot JM. Impact of time from symptom onset to puncture, and puncture to reperfusion, in endovascular therapy in the late time window (> 6 h). Int J Stroke. 2025;20:357–66. 39501524 10.1177/17474930241300073

[15] Joundi RA Smith EE Ganesh A. Time from hospital arrival until endovascular thrombectomy and patient-reported outcomes in acute ischemic stroke. JAMA Neurol. 2024;81:752–61. 38829660 10.1001/jamaneurol.2024.1562 PMC11148789

[16] Chen CH Bala F Najm M. Effect of needle-to-puncture time on reperfusion outcome in acute ischemic stroke. Cerebrovasc Dis. 2024;53:168–75. 37494909 10.1159/000532118

[17] Kalmar PJ Tarkanyi G Karadi ZN Szapary L Bosnyak E. The impact of diabetes mellitus and admission hyperglycemia on clinical outcomes after recanalization therapies for acute ischemic stroke: STAY ALIVE national prospective registry. Life (Basel). 2022;12:632. 35629300 10.3390/life12050632 PMC9147213

[18] Yan J Huang J Pu T. Association of admission hyperglycemia with clinical outcomes in patients with symptomatic intracranial hemorrhage after endovascular treatment for large vessel occlusive stroke. Clin Interv Aging. 2024; Volume19:1545–56. 39347479 10.2147/CIA.S453389 PMC11430306

[19] Genceviciute K Göldlin MB Kurmann CC. Association of diabetes mellitus and admission glucose levels with outcome after endovascular therapy in acute ischaemic stroke in anterior circulation. Eur J Neurol. 2022;29:2996–3008. 35719010 10.1111/ene.15456 PMC9544025

[20] Chang Y Lai PC Huang CY. Tranexamic acid in spontaneous intracerebral hemorrhage: a meta-analysis. Eur J Med Res. 2025;30:133. 40001238 10.1186/s40001-025-02385-x PMC11854317

[21] Jitpratoom P Boonyasiri A. Factors associated with an increased risk of developing pneumonia during acute ischemic stroke hospitalization. PLoS One. 2024;19:e0296938. 38198494 10.1371/journal.pone.0296938 PMC10781189

[22] Zhang P Chen L Ye XF. Outcome and risk of poststroke pneumonia in patients with acute ischemic stroke after endovascular thrombectomy: a post hoc analysis of the DIRECT-MT trial. Neurocrit Care. 2024;41:489–97. 38480608 10.1007/s12028-024-01947-x

[23] Liu XC Chang XJ Zhao SR. Identification of risk factors and construction of a nomogram predictive model for post-stroke infection in patients with acute ischemic stroke. World J Clin Cases. 2024;12:4048–56. 39015898 10.12998/wjcc.v12.i20.4048 PMC11235550

[24] Liu G Zhu H Li L. Effectiveness of collateral status on clinical outcomes in patients with established large infarct: a prospective cohort study. Int J Surg. 2025;111:9342–52. 40844268 10.1097/JS9.0000000000003162 PMC12695354

[25] Rodrigues GM Mohammaden MH Haussen DC. Ghost infarct core following endovascular reperfusion: a risk for computed tomography perfusion misguided selection in stroke. Int J Stroke. 2022;17:897 – 905. 34796765 10.1177/17474930211056228

[26] Zhou Y Zhang L Ospel J. Association of intravenous alteplase, early reperfusion, and clinical outcome in patients with large vessel occlusion stroke: post hoc analysis of the randomized DIRECT-MT trial. Stroke. 2022;53:1828–36. 35240861 10.1161/STROKEAHA.121.037061

