# Supplementary material for: Development and internal validation of a nomogram for predicting 90-day poor functional outcome after mechanical thrombectomy in very elderly patients: A retrospective cohort study
Source: Medicine (Baltimore). 2026 Aug 21;105(34):e50265. doi: 10.1097/MD.0000000000050265 (PMC13502768; doi:10.1097/MD.0000000000050265)
Supplement: Supplementary file 1 [file medi-105-e50265-s001.docx]

**Table S1. Full specification of the final prediction model, including intercept and regression coefficients (Firth penalized logistic regression).**

| **Predictor** | **β** | **SE** | **OR** | **OR (95% CI)** | **p value** |
| --- | --- | --- | --- | --- | --- |
| NIHSS at admission | 0.1719 | 0.0455 | 1.188 | 1.086–1.299 | **0.0002** |
| Diabetes | 0.3401 | 0.4025 | 1.405 | 0.638–3.093 | 0.3981 |
| Puncture-to-recanalization time (min) | 0.0138 | 0.0081 | 1.014 | 0.998–1.030 | 0.0862 |
| Postoperative pneumonia | 1.1451 | 0.4019 | 3.143 | 1.430–6.908 | **0.0044** |
| Symptomatic intracranial hemorrhage | 2.1856 | 0.5676 | 8.896 | 2.925–27.058 | **0.0001** |
| Poor collateral (ASITN/SIR 0–1) | 4.6289 | 1.4959 | 102.404 | 5.458–1921.497 | **0.0020** |

β = regression coefficient; SE = standard error; OR = odds ratio; CI = confidence interval; NIHSS = National Institutes of Health Stroke Scale; ASITN/SIR = American Society for Interventional and Therapeutic Neuroradiology/Society for Interventional Radiology.
