# Supplementary material for: Development and internal validation of a nomogram for predicting 90-day poor functional outcome after mechanical thrombectomy in very elderly patients: A retrospective cohort study
Source: Medicine (Baltimore). 2026 Aug 21;105(34):e50265. doi: 10.1097/MD.0000000000050265 (PMC13502768; doi:10.1097/MD.0000000000050265)
Supplement: Supplementary file 2 [file medi-105-e50265-s002.docx]

**Table S2. Discrimination performance of the prediction model.**

| **Metric** | **Estimate** | **95% CI** | **Notes** |
| --- | --- | --- | --- |
| AUC | 0.8736 | 0.811–0.929 | Bootstrap percentile CI (B=2000) |
| Optimal cutoff (Youden index) | 0.468 | — |  |
| Sensitivity | 0.7625 | 0.663–0.853 | At cutoff 0.468; bootstrap percentile CI |
| Specificity | 0.9552 | 0.916–0.986 | At cutoff 0.468; bootstrap percentile CI |
| Positive predictive value (PPV) | 0.910 | 0.815–0.966 | At cutoff 0.468 |
| Negative predictive value (NPV) | 0.871 | 0.806–0.920 | At cutoff 0.468 |
| Confusion matrix | TP=61, FP=6, FN=19, TN=128 | — | At cutoff 0.468 |
| Accuracy | 0.883 | — | At cutoff 0.468 |

AUC = area under the receiver operating characteristic curve; CI = confidence interval; PPV = positive predictive value; NPV = negative predictive value; TP = true positive; FP = false positive; FN = false negative; TN = true negative; B = number of bootstrap resamples.
