# Supplementary material for: Development and internal validation of a nomogram for predicting 90-day poor functional outcome after mechanical thrombectomy in very elderly patients: A retrospective cohort study
Source: Medicine (Baltimore). 2026 Aug 21;105(34):e50265. doi: 10.1097/MD.0000000000050265 (PMC13502768; doi:10.1097/MD.0000000000050265)
Supplement: Supplementary file 3 [file medi-105-e50265-s005.docx]

**Table S3. Apparent calibration statistics of the prediction model.**

| **Metric** | **Estimate** | **Notes** |
| --- | --- | --- |
| Brier score | 0.112 |  |
| Calibration intercept | 0.057 |  |
| Calibration slope | 1.090 |  |
| Hosmer–Lemeshow χ² (df=8) | 46.37 |  |
| Hosmer–Lemeshow p value | **2.02×10⁻⁷** |  |

Data are presented as apparent calibration metrics derived from the development cohort. χ² = chi-square.
