# Supplementary material for: Development and internal validation of a nomogram for predicting 90-day poor functional outcome after mechanical thrombectomy in very elderly patients: A retrospective cohort study
Source: Medicine (Baltimore). 2026 Aug 21;105(34):e50265. doi: 10.1097/MD.0000000000050265 (PMC13502768; doi:10.1097/MD.0000000000050265)
Supplement: Supplementary file 4 [file medi-105-e50265-s006.docx]

**Table S4. Decision curve analysis (DCA) summary.**

| **Item** | **Result** |
| --- | --- |
| Threshold probability range with positive net benefit (vs treat-all and treat-none) | 0.03–0.05; 0.15–0.98 |
| Maximum net benefit (pt 0.01–0.98) | pt≈0.01, NB≈0.368 |
| Peak net benefit (pt 0.10–0.60) | pt≈0.11, NB≈0.270 |

DCA = decision curve analysis; NB = net benefit; pt = threshold probability.
