# Supplementary material for: Development and internal validation of a nomogram for predicting 90-day poor functional outcome after mechanical thrombectomy in very elderly patients: A retrospective cohort study
Source: Medicine (Baltimore). 2026 Aug 21;105(34):e50265. doi: 10.1097/MD.0000000000050265 (PMC13502768; doi:10.1097/MD.0000000000050265)
Supplement: Supplementary file 5 [file medi-105-e50265-s008.docx]

**Table S5. Risk stratification by quartiles of predicted risk.**

| **Quartile** | **n** | **Events** | **Event rate** | **Median predicted risk** |
| --- | --- | --- | --- | --- |
| Q1 | 54 | 10 | 18.5% | 0.070 |
| Q2 | 53 | 2 | 3.8% | 0.146 |
| Q3 | 53 | 16 | 30.2% | 0.372 |
| Q4 | 54 | 52 | 96.3% | 0.935 |

Event rate is presented as n/N (%), and median predicted risk is presented as median probability.
